# Engineering Linker-Enhanced OmpG Nanopores for Rapid Single-Molecule Protease Detection

**DOI:** 10.3390/s25216681

**Published:** 2025-11-01

**Authors:** Minji Kim, Bach Pham

**Affiliations:** 1 Food Science Department, University of Massachusetts Amherst, Amherst, MA 01003, USA; 2 Research Center for Marine Integrated Bionics Technology, Pukyong National University, Busan 48513, Republic of Korea; 3 Department of Chemistry, University of Science, Vietnam National University, Hanoi 11000, Vietnam

**Keywords:** nanopore sensor, outer membrane protein G (OmpG), single-molecular sensing, single-channel recording, peptide linker, cleavage efficacy

## Abstract

**Highlights:**

**What are the main findings?**

- OmpG nanopores enable sensitive single-molecule detection of protease activity.
- Loop length and charge modifications via linkers boost substrate accessibility.

**What are the implications of the main finding?**

- Dual linkers enhance substrate accessibility and cleavage efficiency in nanopores.
- Engineered OmpG provides a versatile platform for protease biosensing applications.

**Abstract:**

Single-molecule nanopore sensors have enabled real-time detection of enzymatic cleavage events, yet achieving sensitive and specific analysis of protease activity remains an important challenge for diagnostic applications. We engineered OmpG nanopore constructs incorporating thrombin recognition peptides into loop 6 with varied flexible and negatively charged linkers to optimize accessibility and cleavage. SDS-PAGE gel analysis showed that constructs with the recognition peptide placed after residue D225 and incorporating dual linkers achieved cleavage efficiencies up to 95%, whereas constructs without linkers showed limited cleavage. Single-channel recordings revealed that linker integration modulates pore conductance, with extended loops exhibiting intermediate open-state currents near 18 pA compared to 25 pA in wild-type OmpG. Upon thrombin addition, rapid and irreversible current drops confirmed real-time protease activity detection. These results demonstrate the critical role of linker design, particularly flexibility and charge, in optimizing nanopore protease sensors, providing a versatile platform for biomedical applications.

## 1. Introduction

Nanopore sensing technology has emerged as a powerful technique for single-molecule analysis by measuring ionic current changes as individual biomolecules interact with or translocate through a nanosized pore [1]. The ability to distinguish individual molecules using protein nanopores was first established when researchers showed polymer residence times in the pore were much longer than expected, laying the foundation for DNA sequencing and polymer analysis [2,3]. Nanopore detection methods have been widely applied in diverse fields, including DNA sequencing [4,5,6] and in a broad range of substance detection processes such as small molecules [7,8], polymers [9,10], peptides [11,12,13], and proteins [14,15,16]. Biological nanopores, such as α-hemolysin (α-HL), Mycobacterium smegmatis porin A (MspA), Fragaceatoxin C (FraC), phi29 DNA packaging motor protein, cytolysin A (ClyA), pleurotolysin (PlyAB), cytolysin Perfringolysin O (PFO), ferric hydroxamate uptake component A (FhuA), bacterial curli transport lipoprotein (CsgG), and outer membrane protein G (OmpG), offer reproducible structures with well-defined pores, making them attractive scaffolds for engineering biosensors [17,18].

Among biological nanopores, OmpG from *Escherichia coli* is notable for its monomeric β-barrel architecture, comprising 14 strands and seven flexible extracellular loops [19,20,21,22,23]. This monomeric structure allows for efficient genetic and chemical modification, providing flexibility for biosensor engineering and offering distinct advantages over multimeric nanopores in biomedical applications [23]. Loop 6 plays an important role in gating by undergoing conformational changes that control access to the pore lumen [24]. The extracellular loops can be engineered to display affinity ligands. Once analytes are captured by these ligands, they interact with OmpG loops, causing structural alterations in loop conformational dynamics, which generate distinctive current gating patterns that enable the detection of the analytes.

Detecting protease activity at the single-molecule level has become a powerful approach in biomedical research and diagnostics, offering more sensitivity than traditional bulk assays such as colorimetric/fluorometric methods, antibody detection-based assays, and mass spectroscopy [25]. Proteases regulate multiple pathological processes, including cancer progression, neurodegenerative disease, and inflammation [26]. Because protease activity is often spatially and temporally regulated, traditional bulk assays often fail to capture the heterogeneity and transient nature of protease function in complex biological environments. Single-molecule protease detection enables direct observation of enzyme kinetics, substrate specificity, and activity under near-physiological conditions [27].

Thrombin, a serine protease with a molecular mass of approximately 37 kDa, plays an important role in biology as the key enzyme in blood coagulation and platelet aggregation [28]. Beyond its pivotal coagulation function, thrombin is also involved in cellular signaling, tissue repair, inflammation, and regulation of vascular function [29,30]. Abnormal thrombin activity is implicated in a range of pathologies, including thrombosis, cardiovascular disease, and disorders of hemostasis [31,32,33]. Given its broad and critical roles in physiology and disease, developing methods to sensitively detect thrombin activity is highly relevant. Single-molecule approaches enable real-time observation of substrate recognition, under near-physiological conditions, which are essential for advancing both fundamental research and clinical diagnostics.

Previously, our study has demonstrated the use of OmpG engineered with a short, negatively charged recognition peptide for specific protease detection, distinct from other proteases [15]. The engineered recognition peptide showed concentration-dependent signals, while its short length and negative charge had a minimal impact on OmpG’s folding and gating properties. Based on these findings, we aim to further challenge OmpG with recognition peptides of varying length and physicochemical properties to expand its applicability for protease sensing.

In this study, we engineered OmpG nanopores incorporating thrombin recognition peptides, a neutral recognition peptide, within loop 6, employing flexible and charged linker designs to modulate substrate accessibility and cleavage efficiency. By varying linker properties and insertion sites, we demonstrated how structural factors can influence protease recognition and the extent of cleavage among different OmpG constructs.

## 2. Materials and Methods

### 2.1. Materials

Primers for OmpG modification were purchased from Eurofins (Louisville, KY, USA). The *E. coli* DH10β and BL21 (DE3) strains were obtained from Thermo Scientific (Waltham, MA, USA). New England Biolabs (Ipswich, MA, USA) provided the 5X Phusion HF buffer, dNTP mixture, DMSO, and Taq polymerase. Plasmid extraction kits were acquired through CoWin Biosciences (Cambridge, MA, USA). The Q Sepharose Fast Flow resin was supplied by Cytiva (Marlborough, MA, USA). Octyl-β-D-Glucopyranoside (OG) was sourced from Chem-Impex (Wood Dale, IL, USA), and Teflon film was ordered from Goodfellow (Pittsburgh, PA, USA). Bovine Thrombin was purchased from BioPharm Laboratories LLC (Bluffdale, UT, USA). Isopropyl β-D-1-thiogalactopyranoside (IPTG), 4-(2-hydroxyethyl)-1-piperazineethanesulfonic acid (HEPES), urea, and Tris were purchased from Boston Bioproducts (Milford, MA, USA). 1,2-diphytanoyl-sn-glycero-3-phosphocholine (DPhPC) was obtained from Avanti Polar Lipids (Alabaster, AL, USA). Other chemicals not mentioned above were ordered from Research Products International (Mt Prospect, IL, USA).

### 2.2. Cloning of OmpG Constructs

The thrombin recognition peptide (GLVPR|GS) was introduced before (OmpG*^Thr^*^1^) and after (OmpG*^Thr2^*) aspartic acid 224 by site-directed mutagenesis PCR, using the plasmid pT7-OmpG*^WT^* as the template. The OmpG*^Thr2^* constructs were further used as the template for making other OmpG constructs with linkers before and after the thrombin recognition peptide. The OmpG variants utilized for single-channel current analysis were generated by introducing an extra PCR procedure to eliminate the signal sequence. Following amplification, the PCR fragments underwent overnight Dpnl restriction digestion at 37 °C to degrade the template plasmid. The digested products were then introduced into competent DH10β cells, which were subsequently plated on LB agar containing 100 µg/mL ampicillin for selection. The presence of mutations in the plasmid inserts was verified by Sanger sequencing performed by Eurofins Genomics.

### 2.3. OmpG Nanopore Construct Preparation

OmpG expression and purification were performed by following the previously described procedure with modifications [34]. Chemically competent *E. coli* BL21 (DE3) were transformed with the OmpG constructs and then cultured in 1 L of 2xYT medium supplemented with 150 μg/mL ampicillin at 37 °C and shaken at 250 rpm. Upon reaching an OD 600 of 0.5, the expression of the target protein was induced by adding 0.5 mM of IPTG. Cells were further incubated at 16 °C and 250 rpm for 16 h. Bacterial cells were collected by centrifugation at 4 °C and 3184 rcf for 20 min. Pellets were resuspended in buffer containing 50 mM Tris-HCl (pH 8.0) and 1 mM EDTA and then lysed by sonication on ice for 14 min. After lysate extraction, a centrifugation step at 20,000 rcf for 20 min at 4 °C was performed. The pellet was mixed with buffer (1.5 M Urea, 50 mM Tris-HCl, pH 8.0) and stirred continuously at 23 °C for 15 min. Further centrifugation isolated inclusion bodies, which were subsequently dissolved in denaturing buffer (8 M Urea, 50 mM Tris-HCl, pH 8.0). After another centrifugation, the supernatant with solubilized OmpG was applied to a 15 mL Q Sepharose-Fast-Flow column under gravity. The column was washed with buffer (8 M Urea, 50 mM Tris-HCl pH 8.0, 75 mM NaCl), and OmpG proteins were eluted in the same buffer with 200 mM NaCl. Purified OmpG was refolded by combining with refolding buffer (110 mM OG, 50 mM Tri-HCl, pH 9.0) at a 3:8 volume ratio and then incubated at 37 °C for three days. Refolding efficiency was determined via heat-modifiable gel shifting using 12% SDS-PAGE [35,36]. The final refolded OmpG protein preparations were kept at −80 °C in 20% glycerol for storage.

### 2.4. Cleavage Assay of OmpG Proteins by Thrombin

Thrombin was added to the refolded OmpG constructs with a molar ratio of 1:5. A higher thrombin concentration was selected compared to literature conditions [37,38] to accelerate the cleavage reaction and maximize digestion within the assay timeframe. The mixture was diluted with the same volume of buffer (150 mM NaCl, 50 mM Tris-HCl pH 8.0), followed by a 60 min incubation period at 23 °C. After incubation, all samples were heated at 95 °C for 15 min with the SDS-PAGE sample buffer, before loading to 12% SDS-PAGE gel.

### 2.5. Single-Channel Recording of OmpG Constructs

Single-channel current recordings were performed following the method used by Kim et al. [39]. Briefly, a two-chamber apparatus separated by a Teflon film with a 100 μm aperture was used, pretreated with hexadecane/pentane. Both chambers were filled with buffered solution (300 mM KCl, 50 mM Na_2_HPO_4_, pH 6.0), and DPhPC in pentane was applied to form the lipid bilayer by pipetting across the aperture. Ag/AgCl electrodes were inserted with the cis side grounded. OmpG was added to the cis chamber, and 250 mV was applied until pore insertion and then lowered to 50 mV for signal recording. Thrombin was added to the chamber where the extracellular loops of OmpG*^Thr^* were located; then, the solution in that chamber was stirred for around 15 s. In this experiment, we defined a positive potential when the potential of the chamber where the periplasmic terminals were exposed was positive. Recordings were collected using Axopatch 200B and Digidata 1320A/D (Axon Instruments, Union city, CA, USA), filtered at 2 kHz, and analyzed in Clampfit 11.2 after 500 Hz filtration.

## 3. Results

### 3.1. Design of OmpG Constructs to Detect Active Thrombin

**Figure 1:**
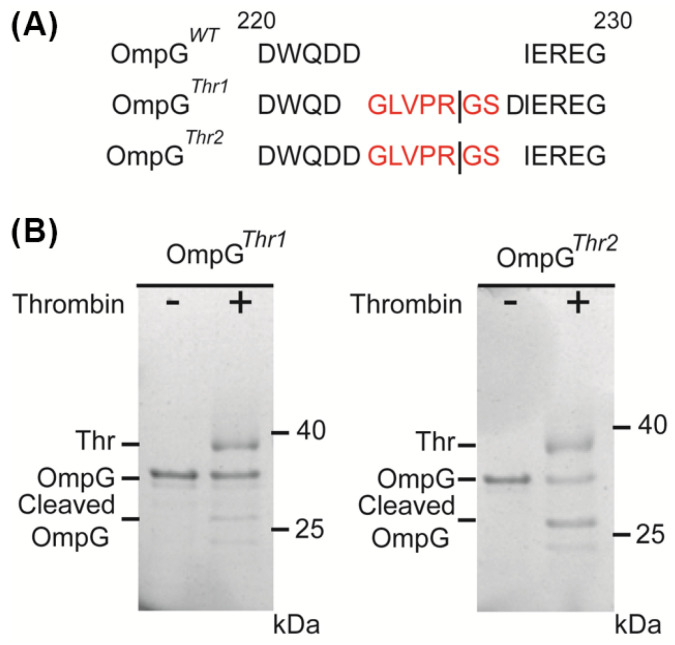
Design of OmpG constructs to detect thrombin. (**A**) The loop 6 sequence alignment of OmpG*^WT^*, OmpG*^Thr1^*, and OmpG*^Thr2^*. The line represents the theoretical thrombin cleavage site. Thrombin recognition peptides are colored in red. (**B**) SDS-PAGE analysis of the cleavage of two OmpG constructs by thrombin. The same amount of OmpG constructs was loaded in each lane. The cleavage efficiency was calculated by comparison of the loss in the intensity of the intact OmpG bands that were analyzed by ImageJ version 1.51.

To evaluate the applicability of the OmpG nanopore design for various proteases, thrombin (Thr) was chosen as a target with modified constructs. Two variants were engineered: OmpGThr1 with a thrombin recognition sequence (GLVPR|GS) inserted before D224 residues in loop 6 of OmpG*^WT^*, and OmpG*^Thr2^* with the same sequence inserted after D225 (Figure 1A). Both OmpG constructs were incubated with thrombin at a 5:1 molar ratio (60 min at 23 °C) and analyzed using SDS-PAGE. Interestingly, we observed that OmpGThr1 was inefficiently cleaved by thrombin, with around 28%, while OmpG*^Thr2^* showed better cleavage efficiency of around 62.3% (Figure 1B). This result reveals that the insertion site critically influences proteolytic cleavage.

**Figure 2:**
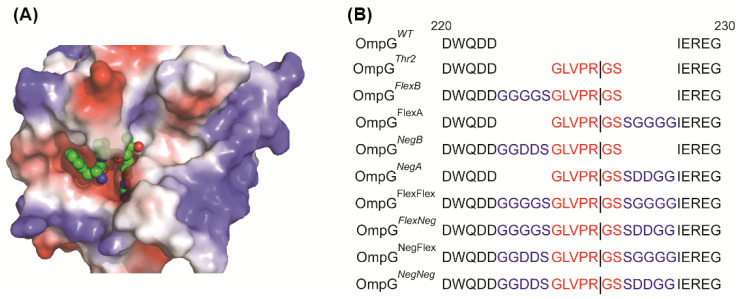
Design of various linkers inserted in OmpG*^Thr2^* construct. (**A**) The electrostatic surface potential of thrombin with a recognition peptide (PDB: 1PPB). The Tyr-Pro-Pro-Trp peptide is shown in balls. (**B**) The loop 6 sequence alignment of OmpG*^WT^* and OmpG*^Thr2^* and eight linker constructs. The sequences of the thrombin recognition peptides are shown in red, and the linkers are shown in blue.

The thrombin structure shows that the binding pocket of thrombin is quite deep (Figure 2A). Thus, we hypothesized that steric hindrance from OmpG’s loops may restrict thrombin’s access to the recognition site in loop 6. To address this, we modified OmpG*^Thr2^* with different linkers to extend the distance between the recognition sequence and OmpG to improve cleavage efficiency. Also, a large positive surface charge near the active site, based on Poisson-Boltzmann equation calculations modeling the interaction of the protein’s fixed charges with the solvent and ions, was observed (Figure 2A). Therefore, the effect of the linker charge was investigated. There are two types of linkers: a neutral and flexible linker (Flex, GGGGS) and a negatively charged linker (Neg, GGDDS). These linkers can be placed either before (FlexB and NegB) or after (FlexA or NegA) the thrombin’s recognition peptide. All the constructs are shown in Figure 2B.

**Figure 3:**
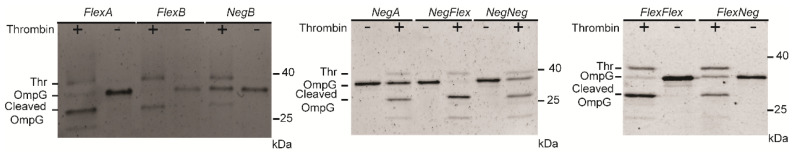
Representative SDS-PAGE gels showing the cleavage of eight OmpG constructs by thrombin. The same amount of OmpG constructs was loaded in each lane. The mole ratio of OmpG to thrombin was 5:1, and the mixture was incubated for 1 h at 23 °C. The buffer used in the cleavage assay was 300 mM NaCl, 50 mM Tris-HCl buffer, pH 8.0.

Cleavage assays at the same molar ratio and conditions demonstrated varied efficiencies across constructs. The reported cleavage efficiencies reflect results from a single experimental dataset (Figure 3). Constructs with flexible linkers showed better cleavage efficiency compared to OmpG*^Thr2^*, supporting our hypothesis that linker flexibility facilitates proper alignments of the peptide within the thrombin binding pocket. Otherwise, the negative linker may enhance the interaction between thrombin and OmpG, but the linker is more rigid, so thrombin cannot bind the recognition peptide in the right conformation for effective cleavage. Moreover, the flexibility of OmpG loop 6 may be a critical factor for thrombin cleavage. Although both single-negative linkers showed low cleavage efficiency of around 40%, the double-negative linker exhibited a 2-fold increase in cleavage efficiency of around 72% (Table 1). These results highlight the importance of loop 6 flexibility and recognition peptide conformation in enabling thrombin cleavage, emphasizing that adaptability is crucial given the enzyme’s deep binding site.

### 3.2. Single-Molecule Detection of Active Thrombin by Designed OmpG Constructs

**Figure 4:**
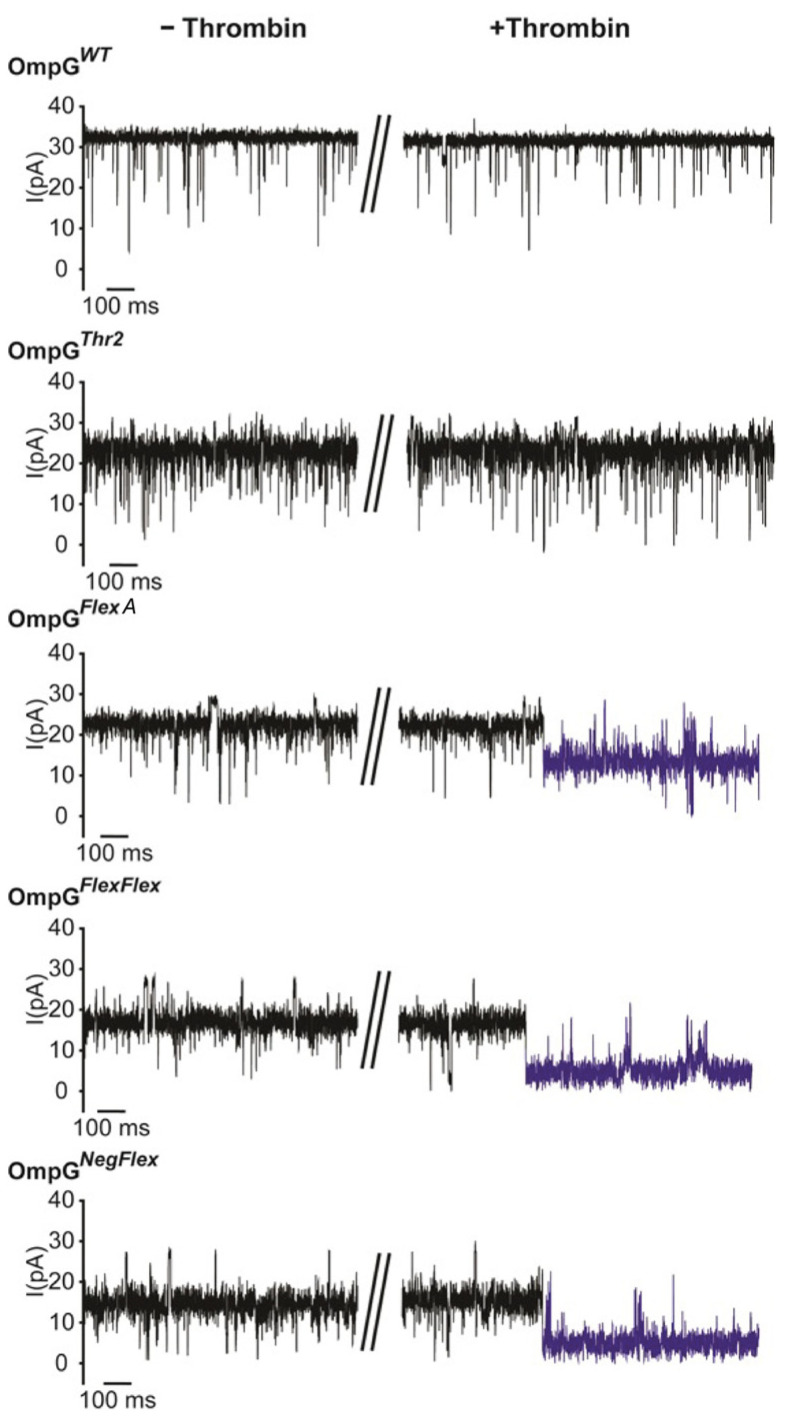
Representative single-channel recording traces of OmpG constructs before and after adding thrombin. The post-cleavage event is highlighted in blue. The final concentration of thrombin was 0.2 µM. The buffer was 300 mM KCl 20 mM Tris HCl pH 8.0 and the applied voltage was 50 mV. The collapsed section of the data trace correspond to the time point at which thrombin was added.

To evaluate the effect of linker engineering on protease sensing, we examined OmpG*^Thr2^*, OmpG*^FlexA^*, OmpG*^FlexFlex^*, and OmpG*^NegFlex^* constructs using single-channel recordings (Figure 4). Modifications that introduced flexible or charged linkers to loop 6 did not adversely impact OmpG refolding or its ability to gate, and all constructs, including those with bulky or negatively charged residues, exhibited stable open and closed states compared to the wild-type OmpG. However, constructs with extended loop regions showed reduced maximum open-pore currents, exhibiting an intermediate conductance of about 18 pA compared to approximately 25 pA for the wild type, suggesting structural adaptation as a result of linker integration (Figure 4).

Significantly, the presence and character of the inserted linker were decisive for protease accessibility and cleavage efficiency. Introduction of thrombin at a final concentration of 0.2 µM which is comparatively high compared to other target concentrations in OmpG studies to triggered rapid and irreversible current drops in OmpG*^FlexA^*, OmpG*^FlexFlex^*, and OmpG*^NegFlex^* constructs. Both double-linker constructs were cleaved within 10 min, while the FlexA construct took 2 h. Otherwise, OmpG*^Thr2^*, lacking linker extension, remained uncleaved after 16 h, which agrees with the previous gel cleavage analysis. For OmpG*^FlexA^*, we observed that the current of OmpG decreased to 54 ± 4%, from 23 ± 2 pA to 12 ± 3 pA (*n* = 3). On other hand, the open-pore currents of both OmpG*^FlexFlex^* and OmpG*^NegFlex^* decreased to 26 ± 5% (*n* = 3) after thrombin cleavage. The cleavage signals were irreversible (Figure 4, highlighted in blue) due to thrombin cleaving the loop.

These findings highlight that flexible linkers dramatically enhance cleavage efficiency by improving recognition peptide accessibility and conformational alignment within the OmpG binding pocket. Charged, rigid linkers, while less effective than their flexible counterparts, still promote proteolysis compared to constructs without extension. This highlights linker engineering as a powerful strategy to single-molecule sensing specificity and efficiency, positioning OmpG nanopores as highly adaptable platforms for protease detection and more broadly, engineered biosensing applications.

## 4. Discussion and Conclusions

This study investigated how the chemical nature and the insertion position of protease recognition peptides within the OmpG nanopore loop influence protease sensing efficiency and single-molecule detection by combining biochemical assays and electrophysiological recordings. First, to determine which position and length of OmpG loop 6 would provide better cleavage efficiency, we used thrombin as a target and varied the linker type (negative charged or neutral) between the OmpG native loop and thrombin recognition peptide. Although negatively charged linkers enhance charge-based interactions with positively charged enzyme pockets, their inherent rigidity can limit substrate conformational freedom, leading to less optimal cleavage compared to flexible linker designs. This phenomenon was also observed in studies using supercharged polypeptide-modified nanopores [40]. Nonetheless, the synergistic combination of flexible and charged linkers demonstrated robust improvements as both cleavage yields and signal reliability were maximized. This suggests that future biosensor development may benefit from combinational linker engineering strategies. Furthermore, we confirmed that loop 6 can accommodate diverse recognition peptides, including longer and bulkier sequences, without compromising OmpG refolding or gating characteristics in single-channel recordings.

Like numerous previous studies, such as the detection of RNase activity using alpha-hemolysin nanopores [3], the demonstrated ability to detect protease activity in real-time at the single-molecule level continues to hold significant potential for biomedical and diagnostic applications. Proteases, like thrombin, play critical roles in blood coagulation and are biomarkers for many pathological conditions [41,42]. The OmpG nanopore platform offers a label-free and highly sensitive method for monitoring protease activity that could be integrated into point-of-care diagnostic devices [36]. Furthermore, the use of linkers and recognition peptide design allows for the adaptation of this sensor to detect other clinically relevant proteases or enzymes, broadening its potential medical applications.

This study highlights that both the position of peptide insertion within loop 6 and the properties of linkers are critical determinants of effective protease recognition and cleavage. Lengthening loop 6 with dual linkers enhances the accessibility of the peptide without compromising nanopore stability or gating properties. This is important as loop 6 of OmpG plays a crucial role in generating the gating effect on current recordings [43].

A primary limitation is that all thrombin detection experiments were conducted at a fixed, relatively high concentration to promote rapid and robust capture events in the OmpG nanopore system. According to diffusion-controlled ligand binding theory [44,45,46], however, quantitative metrics such as first capture time are expected to vary predictably with analyte concentration. While our results at high thrombin concentration align with these kinetic models, future studies should systemically vary thrombin concentrations to validate this relationship and construct calibration curves. Such experiments would further strengthen the relevance of the OmpG nanopore biosensor for sensitive and reliable biomarker detection in clinical diagnostics.

In summary, this work confirms that linker engineering is a vital strategy for overcoming steric and electrostatic barriers to protease detection in nanopore sensors. It establishes an adaptable, sensitive, and practical framework for next-generation biomolecular sensing with wide-reaching biomedical and diagnostic applications. Continued advancement should focus on expanding the repertoire of recognition peptides tailored for diverse clinically relevant proteases, optimizing linker chemistry for enhanced specificity, and integrating multiplexed sensing capabilities. The multiplexing capability of the OmpG nanopore was demonstrated in our previous study [47], and the current study can further extend these findings.

## Figures and Tables

**Table 1:** Gel cleavage quantification. The cleavage efficiency was calculated by comparing the loss in the intensity of the intact OmpG bands.

	Cleavage Efficiency (%)
OmpG*^Thr2^*	62.3
OmpG*^FlexA^*	90.7
OmpG*^FlexB^*	86.3
OmpG*^NegB^*	34.1
OmpG*^NegA^*	37.3
OmpG*^NegFlex^*	95.7
OmpG*^NegNeg^*	72.1
OmpG*^FlexFlex^*	93.9
OmpG*^FlexNeg^*	70.7

## Data Availability

The original contributions presented in this study are included in the article. Further inquiries can be directed to the corresponding author.
